# Alcohol and Other Drug Use during Pregnancy among Women Attending Midwife Obstetric Units in the Cape Metropole, South Africa

**DOI:** 10.1155/2014/871427

**Published:** 2014-02-03

**Authors:** Petal Petersen Williams, Esmé Jordaan, Catherine Mathews, Carl Lombard, Charles D. H. Parry

**Affiliations:** ^1^Alcohol & Drug Abuse Research Unit, South African Medical Research Council, P.O. Box 19070, Tygerberg 7505, South Africa; ^2^Biostatistics Unit, South African Medical Research Council, P.O. Box 19070, Tygerberg 7505, South Africa; ^3^Statistics and Population Studies Department, University of the Western Cape, P.O. Box X17, Bellville 7535, South Africa; ^4^Health Systems Research Unit, South African Medical Research Council, P.O. Box 19070, Tygerberg 7505, South Africa; ^5^Women's Health Research Unit, School of Public Health and Family Medicine, University of Cape Town, Falmouth Building, Medical Campus, Observatory 7925, South Africa; ^6^Department of Psychiatry, Stellenbosch University, P.O. Box 19063, Tygerberg 7505, South Africa

## Abstract

Little is known about the nature and extent of alcohol and other drug (AOD) use among pregnant women in Cape Town, South Africa, despite the very high levels of AOD use in this part of the country. A cross-sectional survey was conducted among pregnant women attending 11 Midwife Obstetric Units (MOUs) in greater Cape Town. A two-stage cluster survey design was used. In total, 5231 pregnant women were screened to assess self-reported prevalence estimates. Of these, 684 (13.1%) were intentionally subsampled and completed an interviewer-administered questionnaire and provided a urine sample for biological screening. Urinalyses showed that 8.8% (95% CI: 6.7–10.9) of the subsample tested positive for at least one illicit drug. This is higher than the self-reported prevalence (3.6%). In addition, 19.6% (95% CI: 16.3–22.8) of the sub-sample tested positive for alcohol which is lower than the self-reported prevalence (36.9%). There are high levels of substance use among pregnant women attending public sector antenatal clinics. There is a need for routine screening for AOD use and appropriate responses depending on the women's level of risk.

## 1. Introduction

Alcohol and Other Drug (AOD) use during pregnancy negatively impacts both mother and child, and the effects of maternal alcohol and illicit drug use on the fetus and postnatal infant outcomes are growing public health concerns in South Africa and elsewhere [1]. Illicit drug use in pregnancy has been associated with preterm delivery, low birth weight infants, placental abruption, neonatal abstinence syndrome, and neonatal intensive care unit admissions [2]. The most severe consequences of alcohol use during pregnancy are the Fetal Alcohol Spectrum Disorders (FASD) [3]. In South Africa, considerable research has been conducted on FASD and it has been found that the Western Cape in particular has one of the highest known prevalence rates in the world [4]. These authors report that the overall rate of FASD among first-grade children in a wine-growing region in the Western Cape was 135.1 to 207.5 per 1000 (or 13.6 to 20.9%) in the most recent wave of this research. However, little is known about the nature, extent, and impact of other drug use among pregnant women in South Africa. AOD use and the burden of drug use have been shown to be greater in the Western Cape compared with other provinces [5]. Despite this, access to treatment remains low in Cape Town, the capital of the Western Cape [6], and few people receive treatment including women of childbearing age.

Anecdotal evidence from several hospitals and clinics suggests that there is an increasing number of babies being born to substance-abusing mothers, particularly those using methamphetamine in the Western Cape [7, 8]. For example, in 2006, 10 percent of the 100 pregnant participants of a small study at antenatal clinics in the Tygerberg area admitted to using methamphetamine (Dr. B. Vythilingum, personal communication, July 10, 2007). Furthermore, 13 to 15% of pregnant women who took part in a smoking cessation intervention study between 2006 and 2007 in two local areas in the Cape Metropole reported current use of illicit drugs, while 49 to 55% drank alcohol [9]. Both these studies were very small in scope and their main aim was not to investigate the extent of drug use among pregnant women.

Although a study in Midwife Obstetric Units (MOUs) in the Cape Metropole found a high rate of self-disclosure of various substances used during pregnancy [9], substance use in pregnancy remains a controversial and socially undesirable behaviour. Many pregnant women are likely to deny their use of substances for fear of being stigmatized or criticized [10], and several studies have indicated under-reporting of drug use by pregnant women [11–13]. These studies provide evidence that self-disclosure may not be an accurate indication of the extent of substance use, particularly when there is very little time to build rapport in busy, overcrowded, and under-staffed public health facilities. In such settings, biological markers could be paramount in identifying women who use alcohol and other drugs (AODs) to guide appropriate service provision. Although studies in South Africa have tested for cotinine to identify women who smoke nicotine [9], no studies have reported using biological markers to test for illicit drug use and determine prevalence among pregnant women in primary healthcare facilities. The current study was thus undertaken to describe three assessments of AOD use, namely, self-report at the first stage during screening, an assessment at the second stage based on the Alcohol, Smoking, and Substance Involvement Test (ASSIST), and biological urine tests, in order to estimate the prevalence of such use among pregnant women attending public sector antenatal birthing units in the Cape Metropole.

## 2. Methods

### 2.1. Study Setting and Population

The study population comprised pregnant women who attended 11 public sector, community-based clinics (MOUs), scattered across eight health subdistricts, for antenatal care and delivery in the greater Cape Town, a city of over three million people. The majority of the people are dependent on government funded health services. These 11 MOUs were all the MOUs in greater Cape Town and fall under the Western Cape Department of Health. All are found in areas that were classified as “African black” or “Coloured” under the apartheid regime and can be considered as serving previously disadvantaged communities. MOUs are birthing units run by midwives in the community for primary health care patients. A full initial assessment occurs at the first or booking visit where after the pregnancy is monitored through regular follow up visits (antenatal care), babies are delivered by midwives at these facilities and after birth care of the mother and baby also occur. At any stage referral to a hospital can occur, should this be necessary. The combined annual total for women seen in the 11 MOU clinics during 2007 and 2008 was 41715. About 3% of these pregnant women attended the smallest clinic and 17% attended the largest clinic in the area.

### 2.2. Design and Sample Selection

A two-stage cluster survey design was used to collect AOD data among pregnant women attending their first booking visit at any of the 11 MOUs in greater Cape Town. The first booking might occur any time from their first trimester to their third trimester of pregnancy.

The data from the first stage of the survey was used for estimating the initial self-reported alcohol and drug prevalence in the population. Drug prevalence was assumed to be around 3 to 5 percent for the various MOUs, using an inflation factor of around 1.2 for the design effect. Thus, for the prevalence estimation of reported drug use, a sample size of around 5394 was required to produce a two-sided 95% confidence interval with a precision equal to 0.01 when the sample proportion is 0.035.

Proportional sampling allocation was used across the 11 MOUs, using the total clinic attendance by pregnant women in 2007 and 2008 (*N* = 41715) to determine the number of first bookings from each MOU to be screened. The sampling fraction was around 12.5% overall, varying from 10% to 15% for the various MOUs. A further objective was to estimate the prevalence of drug and alcohol use by urinalysis. To achieve this, a second stage subsample was selected from the first stage group of pregnant women, for detailed interviewing about AOD use (ASSIST) and for urine testing. The second stage sampling and subsequent interview and urine collection occurred immediately.

The second stage selection of women from the MOUs was done with unequal probability sampling as follows. The pregnant women already screened were stratified into three mutually exclusive strata according to their reporting at the first stage. Stratum A comprised woman who reported drug use in the first stage; Stratum B comprised woman who reported alcohol use only; and Stratum C comprised women who reported no AOD use. Since it was important to ensure an adequate sample size for the positive drug group, all women belonging to stratum A were selected. For the presumed larger stratums B and C, systematic random sampling techniques were employed. It was planned that from the alcohol only stratum, every 10th observation and from the no AOD use stratum every 5th observation was to be sampled. The choices of 10th and 5th observation were quite arbitrary, although we believed that a certain measure of underreporting would take place and reduce the no AOD group, and hence a larger proportion of women from group C than group B were being recruited.

### 2.3. Measures

Two modes of data collection took place: interviewer-administered questionnaires to collect participants' reports of AOD use and biological measures of AOD use.

#### 2.3.1. Demographics

Data were collected on stage two sub-sample participants' age, race, education, marital status, employment, the number of times they had been pregnant before, whether their current pregnancy was a planned pregnancy, and how advanced the pregnancy was. Participants were asked whether or not they had electricity and certain household items such as a radio, television, telephone, fridge, computer, washing machine, and cell phone. Those who had four or less of these were grouped into a low socioeconomic status (SES) group and those who had five or more were grouped into a high SES group [14].

#### 2.3.2. Self-Reported Substance Use

At stage one, all women attending the clinic for their first/booking visit for their current pregnancy were asked by fieldworkers whether they had consumed alcohol or used drugs during their current pregnancy and/or in the three months before they knew they were pregnant. This screening instrument also asked their age and whether their current visit to the clinic was their first visit for their current pregnancy. The stage two sub-sample (*n* = 684) of women (selected using the procedures described previously) completed the ASSIST. The ASSIST is a brief screening questionnaire consisting of 8 items and was developed by the World Health Organisation (WHO) and an international team of substance use researchers as a simple method of screening for hazardous, harmful, and dependent use of alcohol, tobacco, and other psychoactive substances [15]. Each question on the ASSIST has a set of responses to choose from, and each response has a numerical score. These scores are added together to produce an ASSIST score. A Specific Substance Involvement Score can be obtained for each substance, and it provides a measure of use and problems over the three months prior to the interview and predicts the risk of future substance related problems [15]. Each patient may have up to 10 Specific Substance Involvement Scores depending on how many different types of substances they have used. Patients with ASSIST Specific Substance Involvement scores of three or less (10 for alcohol) are at a lower risk of problems related to the use of the substance involved. Mid-range scores between 4 (11 for alcohol) and 26 for any substance are an indication of hazardous or harmful use of that substance and places the patient at moderate risk of harm, while a score of 27 or higher for any substance suggests that the patient is at high risk of dependence on that substance [15]. In the current research, if a participant used more than one substance in the previous three months, only the highest score was included for the Total Substance Involvement Score.

#### 2.3.3. Urinalysis

Urine samples were tested for alcohol, cannabis, heroin, cocaine, methamphetamine, benzodiazepines, and methaqualone (Mandrax) at a private drug testing laboratory by means of immunoassays. The tests provided a measure of the metabolites of the specific drugs for which participants were tested. Ethyl Glucuronide (EtG) is a direct metabolite of alcohol (ethanol). Its presence in urine may be used to detect recent alcohol consumption, even after ethanol is no longer measurable. Cut-off levels for benzodiazepine, cannabis, cocaine, methamphetamine, heroin, mandrax, and alcohol were 300 ng/mL, 50 ng/mL, 300 ng/mL, 300 ng/mL, 300 ng/mL, 300 ng/mL, and 100 ng/mL, respectively. These cut-off levels were based on standardized threshold concentration levels established by the international regulating authorities. For heroin and methamphetamine, cut-off levels were chosen that corresponded to research previously conducted, 300 ng/mL for heroin [16] and 300 ng/mL for methamphetamine [17]. Research has demonstrated that immunoassay urine screening had high specificity and agreement but variable sensitivity [18]. Immunoassay urine drug screens are however commonly used as they are relatively inexpensive and rapid. For this reason, standard immunoassay testing is the preferred initial test for urine drug screening [19].

### 2.4. Study Procedure

The study was conducted over a period of approximately 100 days from February to March 2010 and again from November 2010 to March 2011. This split occurred in all clinics except one where data collection was completed during the first period. Ten fieldworkers, working either in pairs or threes (depending on the size of the clinic) were able to collect data in four clinics simultaneously and once the sample size was reached in each clinic, they were able to move to the next clinic. Fieldwork had to be suspended for seven months resulting in the split between the two data collection periods. All women provided informed consent to be screened for possible involvement in the stage two survey. To be eligible for participation in this survey, women had to be pregnant, attending the clinic for their first/booking visit, be 16 years or older, and give written consent to participate in the study and provide a urine sample to be tested for AOD. Questionnaires and urine samples were linked using a unique barcode. After completion of the stage two interview, each participant received an incentive for their time and participation in the form of a chain store voucher to the value of R50 (approximately $6). They were also provided with a resource list, with contact numbers, of all institutions within reach where they could go for substance abuse treatment or other personal problems. Ethical approval to conduct the research was obtained from the Faculty of Heath Sciences Research Ethics Committee at the University of Cape Town. Permission to conduct the survey in the MOUs was obtained from the Western Cape Department of Health.

### 2.5. Data Analysis

Appropriate sampling weights were determined according to the study design to generalize the results to the described population of pregnant women.

A survey analysis was conducted, using appropriate weights for the proportional allocation of women, to estimate the reported drug and alcohol prevalence and 95% confidence limits in the screening sample. A finite population correction was also used. Prevalences and standard errors were estimated for the drug group, the alcohol only group, and the no AOD group. The prevalence for the larger group of women using alcohol regardless of drug use was also estimated (alcohol and drug group in Table 2).

For estimating the prevalence reported using the ASSIST and the urinalysis involving the sub-sample, a survey analysis was used, incorporating appropriate weights for the women belonging to the three strata A, B, and C. The proportional clinic weights and the sub-sample weights were combined to obtain the appropriate sampling weights for calculating the prevalence of drug and alcohol use, with standard errors and 95% confidence limits in the sub-sample.

To determine the demographic factors impacting on the prevalence, a survey logistic regression analysis was used with the appropriate weighting as mentioned before. Demographic variables considered were age, race, education, marital status, employment status, socioeconomic status, parity, whether it was a planned pregnancy, and gestational age of the women.

## 3. Results

### 3.1. Characteristics of the Sample

**Figure 1:**
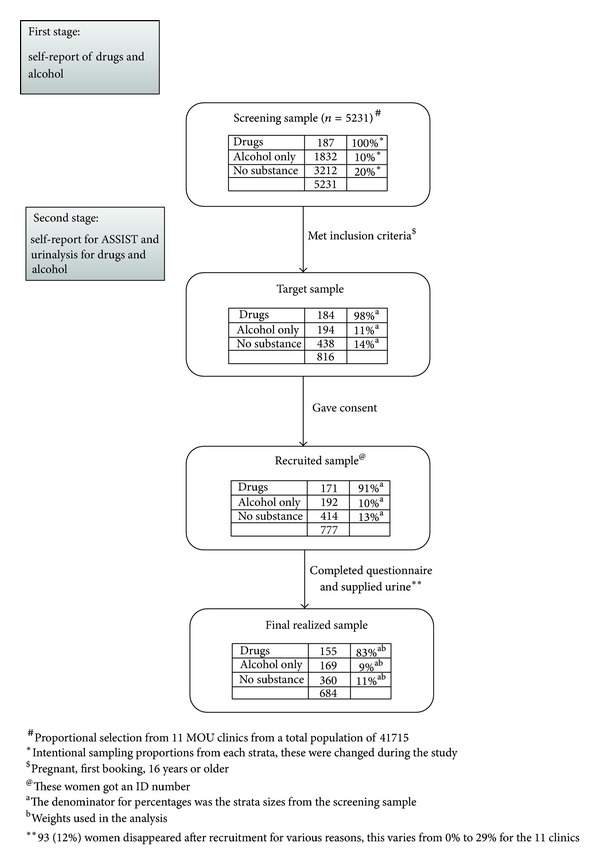


Of the 5231 pregnant women screened, 187 self-reported drug use, 1832 self-reported alcohol use, and 3212 reported no AOD use. Of the 187 drug users, 184 met inclusion criteria with 171 agreeing to participate and willing to provide a urine sample. Of the 1832 alcohol users, every tenth person was selected and 194 met inclusion criteria with 192 agreeing to participate and willing to provide a urine sample. Finally, of the 3212 nonusers, every fifth person was selected and 438 met inclusion criteria with 414 agreeing to participate and willing to provide a urine sample. However, 93 participants did not complete the questionnaire and in so doing reduced the final sample of participants to 684.  Figure 1 illustrates the sample flowchart. The reasons provided by pregnant women for not completing the questionnaire varied and included among other things, feeling tired, hungry, ran out of time, or simply were no longer interested. Of the 93 women, 16 (8.7%) were in the drug group, 23 (12.0%) were in the alcohol only group, and 54 (13.0%) were in the no AOD group. Therefore, compared to the other two groups, a lower proportion of women disappeared from the drug group. The 684 participants had a mean age of 26 years; 60.3% were Black African, 39.1% Coloured, and 1.6% White or Asian. (The terms “White,” “Black,” and “Coloured” refer to demographic markers and do not signify inherent characteristics. They refer to people of European, African, and mixed (African, European, and/or Asian) ancestry, respectively. The continued use of these markers in South Africa is important for monitoring improvements in health and socio-economic disparities, identifying vulnerable sections of the population, and planning effective prevention and intervention programmes.) Ninety-two percent had some secondary education, 66.9% were never married/single, and 56.9% were unemployed. The majority of the participants had five or more items in their homes (75.9%). For the majority of the participants, their current pregnancy was their second pregnancy (35.1%) followed very closely by those for whom the current pregnancy was their first (34.0%). Most women reported that their current pregnancy was unplanned (68.2%) and the mean gestational age at the booking visit was 18.9 weeks with the majority of women booking in their second trimester (57.4%) (Table 1).

### 3.2. Prevalence of Alcohol and Other Drug (AOD) Use

#### 3.2.1. Self-Report in the Screened Sample

Among 5231 pregnant women screened, 36.9% reported that they had consumed alcohol during their current pregnancy or in the three months before they knew they were pregnant (of these 34.9% consumed alcohol only and no other drugs). Far fewer reported drug use (3.6%) (with 1.6% reporting use of drugs only and no alcohol) and 61.6% reported no AOD use at all (Table 2).

#### 3.2.2. ASSIST

The 684 participants recruited completed the ASSIST.  Table 3 shows lifetime use and frequency use in the past three months of tobacco, alcohol, cannabis, and methamphetamine. The reported use of any other drugs was very low and the numbers very small. In total, 39.2% reported lifetime tobacco use (21.6% reported weekly/daily use and 6.7% occasional use), 59.0% lifetime alcohol use (9.9% reported weekly/daily use and 26.9% occasional use), 12.7% lifetime cannabis use (1.2% reported weekly/daily use and 1.7% occasional use), and 6.9% lifetime methamphetamine use (1.3% reported weekly/daily use and 1.1% occasional use).  Table 3 also outlines the participants' level of risk for tobacco, alcohol, cannabis, and methamphetamine. The level of risk associated with the ASSIST measure in the current study is directed at the woman and not necessarily the unborn baby. For all substances, the majority of participants scored in the low-risk category. Twenty-eight percent and 21.9% scored in the medium-risk category for smoking and alcohol respectively, while 3.2% and 2.1% of pregnant women fell in the medium risk category for cannabis, and methamphetamine, respectively.  Table 4 provides the mean Substance Use Involvement Scores among tobacco, alcohol, cannabis and methamphetamine users as well as a total illicit substance involvement score. The mean scores for each of the substances fall within the mid-range scores, indicating a moderate risk of harm for these patients.

#### 3.2.3. Urinalysis

The 684 participants who made up the sub-sample and who completed the full questionnaire also provided a urine sample for biological testing for benzodiazepines, cannabis, cocaine, methamphetamine, heroin, mandrax, and alcohol.

Of the 684 samples collected, 34 specimens were inadvertently destroyed by the drug-testing laboratory before any drug and alcohol testing could be done and another 61 before alcohol and methamphetamine testing could be done. The lost samples were from 7 out of the 11 MOUs. For one MOU, 40% of the samples for alcohol and methamphetamine testing were lost, but this was reduced to 14% of the samples when testing the other drugs. The lost samples represented a similar proportion from the three strata (between 10% and 15% from each stratum). For participants whose urine samples were lost, 31% had self-reported alcohol use and 4% self-reported drug use. For participants with available urine samples, 37% had self-reported alcohol use and 3.7% self-reported drug use. Therefore, it seems that the lost samples did not create any obvious bias in terms of self-reported alcohol (*P* = 0.39) and drug (*P* = 0.89) use at screening.

Urinalyses showed that 18.9% of the samples had a result above 0.0 ng/mL for at least one illicit drug. However, only 8.8% of the urine samples met standardized cut-off criteria to test “positive” for at least one illicit drug (8.1% for methamphetamine, 0% for heroin, 1.8% for cannabis, 0.4% for methaqualone (mandrax), 0% for cocaine, and 0% for benzodiazepine). Additionally, 19.9% of the sample had a result above 0.0 ng/mL for alcohol, and 19.6% of the urine samples met standardized cut-off criteria to test “positive” for alcohol (Table 5).

### 3.3. Impact of Gestational Age and Demographic Variables on the Prevalence

The estimated drug prevalence for women attending their first booking in their last trimester (25+ weeks) was marginally increased compared to the prevalence for women who attended their first booking visit before their third trimester (0–12 weeks: 3.6% (95% CI: 2.3–4.9); 13–24 weeks: 2.9% (95% CI: 2.1–3.6); 25–40 weeks: 5.1% (95% CI: 3.3–7.0)), but this was not statistically significant (*P* > 0.05). The alcohol prevalence was similar for the three trimesters.

Considering the demographic variables, race (*P* = 0.0001; OR Black versus other = 0.12; 95% CI: 0.08–0.20), marital status (*P* = 0.0005; OR married versus other = 0.24; 95% CI: 0.12–0.49), and employment (*P* = 0.0001; OR unemployed versus employed = 4.5; 95% CI: 2.6–7.8) were factors impacting on the drug prevalence. Also, race (*P* = 0.002; OR Black versus other = 0.55; 95% CI: 0.37–0.80) and marital status (*P* = 0.0001; OR married versus other = 0.26; 95% CI: 0.15–0.45) were factors impacting on the alcohol prevalence.

## 4. Discussion

This study found prevalence of drugs among pregnant women as tested by urine analysis to be 8.8%, higher than the self-reported 3.6%. The prevalence of alcohol as tested by urine analysis was 19.6%, which is lower than the self-reported prevalence of 36.9%. Given the similar detection times of alcohol and other drugs in the urine, this finding may indicate that these women may not view alcohol use to be as socially unacceptable as other drug use. They may thus be much more willing to report their use of alcohol while the alcohol had already dissipated from their bodies. Alcohol is also reported to dissipate faster in women compared to men [20]. Compared with previous research conducted in MOUs in the Cape Metro that recorded self-reported drug use of 13% and 15% in the natural history and intervention groups, respectively, and alcohol use of 55% and 49% in the natural history and intervention groups, respectively [9], prevalence in the current study (both self-report and biologically verified) is lower for drug and alcohol use. The current research, however, was a cross-sectional survey. Everett-Murphy et al. conducted an intervention study with a central focus on the provision of social support to pregnant women through peer counselling based on simple brief motivational interviewing (BMI) [9]. This level of support provided by peer counsellors placed in a clinic specifically to deliver an intervention may have made these women more comfortable to disclose AOD use but it is not routinely present in these busy state-run facilities. This difference would have affected only the validity of the self-reported data.

While there are no published data reporting routine screening of pregnant women in antenatal clinics for alcohol and drugs by urine toxicology in South Africa, previous international research (in the United Kingdom and the USA) which used urinalysis to determine prevalence of drug and alcohol use in pregnant women reported prevalence rates ranging between 2.8% and 15.3% [21–24]. Although the women in this study gave informed consent for their urine to be tested for AOD use, they were not informed of this prior to their arrival at the clinic. Therefore, similar to anonymous unlinked studies, the results reported are more likely to be an estimate of true prevalence as patients did not have the chance to abstain from substance use specifically to ensure their urine was negative when tested. However, 93 (12%) participants did not complete the questionnaire nor provided a urine sample and in some cases this may have been to avoid detection of AOD in their urine.

Methamphetamine was found to be the most common illicit drug for which participants tested positive despite a higher cannabis self-report rate. However, alcohol was the most common substance overall. Data from specialist drug abuse treatment centers monitored via the South African Community Epidemiology Network on Drug Use (SACENDU) project have shown that the most common primary substances of abuse reported by the 26 specialist treatment centres/programmes in the Western Cape participating in the project between January and June 2011 were methamphetamine (aka “tik”), followed by alcohol, cannabis, and heroin [25]. Research has further shown that levels of methamphetamine use were higher among pregnant than nonpregnant women while no other substances differed by pregnancy status [26]. Both studies therefore highlight the salience of methamphetamine among patients of childbearing age. In a study conducted in the US, infants who were exposed to methamphetamine prenatally were more likely to exhibit poor suck, to have smaller head circumferences and length, to require neonatal intensive care unit admission, and to be referred to child protective services [27]. This provides further justification for urgently addressing methamphetamine use among pregnant women.

Various methods exist to determine substance use (self-report, urinalysis, meconium tests, hair analysis, and nail analysis). While self-report is the most cost-effective method, relying on self-report alone may not always be adequate in identifying substance use, especially among pregnant women who may not report their substance use to antenatal staff out of fear of their reaction [28]. It has been reported that urine screening remains the most commonly used biomarker despite the limited period during which drugs can be detected [29]. Early and accurate identification of substance-using pregnant women and early intervention could reduce the many adverse pregnancy outcomes associated with maternal substance use. Research on brief interventions, addressing alcohol and tobacco use, points to the efficacy of this method for these substances [30–33]. There is, however, growing evidence of the effectiveness of brief interventions for other substances such as opiates [34], amphetamines [35], benzodiazepines [36], and cannabis [37]. Most of this work has focused on the general population; however, brief interventions with pregnant women have also been shown to be effective [38–41]. Therefore, despite the costs involved in urine toxicology screening, evidence suggests that the benefits outweigh the costs when it comes to testing during pregnancy [42] and then intervening according to the level of risk.

The study findings are subject to the following limitations. Twenty-three percent of the participants were in the first trimester of their pregnancy. Given that the self-report assessments asked about AOD use in the last three months, the possibility exists that AOD use occurred prior to pregnancy for this subset of women. The ASSIST is not a pregnancy-specific screening tool. However, it was designed to screen for substance abuse in primary care settings and pregnant women have been recommended as a target group suitable for an ASSIST screening programme by the developers of this tool [15]. Furthermore, unlike other tools, the ASSIST allows for screening across the spectrum of substances and is relatively easy to administer. More recently, however, it has been argued that its place in pregnancy screening is currently uncertain as it did not uniformly show good agreement with screening tools such as the T-ACE, Revised Fagerstrom Tolerance Questionnaire (RTQ), and the Timeline Follow Back (TLFB), which were selected because they either had been validated in pregnancy or had a history of use with pregnant women [43]. Future research could therefore further investigate the validity of the ASSIST instrument for use in pregnant women. Fieldwork activities were suspended for seven months due to unforeseen circumstances resulting in a split between two data collection periods. Urine samples were collected Mondays to Fridays. If participants used substances over the weekend, it is possible that their urine results will show up as negative, particularly if collected later in the week. It is therefore possible that the prevalence reported here is an underestimation and that the real prevalence is higher. Ninety-five urine samples were not tested for any (*n* = 34) or some (*n* = 61) substances, which accounted for 12.2% of the total samples. However, this did not create any obvious bias in terms of alcohol and drug screening. Future research could focus on collecting data only on a Monday to increase the likelihood of picking up positive results. Other options for biological testing, such as nail analysis, should also be investigated as they may provide a longer detection time. In addition, future research could include biological tests on all first bookings rather than sampling from the larger ‘booking visit' population across sites. Further research is necessary to assess the cost-benefit for routine toxicology screening in primary health care facilities for pregnant women and to assess who would be best placed to conduct routine screening and brief interventions in these facilities.

## 5. Conclusion

Our findings suggest that there are high levels of AOD use among pregnant women attending public sector antenatal clinics in the Cape Metropole. Race, marital status, and employment were demographic factors impacting drug prevalence where being coloured, single, and unemployed were risk factors for drug use. Similarly, race and marital status were demographic factors impacting alcohol prevalence where being coloured and single were risk factors for alcohol use. Pregnant women may not always report their use of alcohol and/or other drugs. However, urine toxicology screening is viable in the clinic setting, and pregnant women are willing to provide urine samples. Study findings support the conclusion that the widespread use of substances (particularly alcohol and methamphetamine) by pregnant (mainly Coloured and Black African) women who access state facilities indicates a need for routine screening of all pregnant women using self-report questionnaires as a minimum and urine testing where possible. This should be followed by brief interventions focused on alcohol and other drug use reductions and cessation where indicated.

## Figures and Tables

**Table 1:** Description of sub-sample participant demographic characteristics (*n* = 684).

	%	SE
Age		
0–19	14.7	1.48
20–29	62.9	2.08
30–39	19.5	1.73
40–49	2.8	0.73
Race		
Black African	60.3	1.70
Coloured	39.1	1.71
White/Asian	1.6	0.55
Education		
Primary	3.5	0.79
Secondary	92.0	1.16
Tertiary—incomplete	1.9	0.62
Tertiary—complete	2.6	0.65
Marital status		
Legally married	18.9	1.65
Traditionally married	7.7	1.15
Living together	4.1	0.84
Never married/single	66.9	2.00
Divorced/separated/widowed	2.4	0.68
Employment status		
Unemployed	56.9	2.14
Employed full-time	23.7	1.86
Employed part-time/self-employed	12.0	1.43
Student	7.4	1.10
Socioeconomic status		
Low (4 or less items)	24.1	1.78
High (5 or more items)	75.9	1.78
Parity		
0	34.0	2.00
1	35.1	2.10
2	19.6	1.70
3	8.2	1.20
≥4	3.1	0.70
Planned pregnancy		
Yes	31.8	2.10
No	68.2	2.10
Gestational age		
0–12 weeks (1st trimester)	22.8	1.90
13–24 weeks (2nd trimester)	57.4	2.30
25–40 weeks (3rd trimester)	19.8	1.80

**Table 2:** Prevalence of self-reported alcohol and other drug use in the screened sample (*n* = 5231).

	*n*	%	SE	95% CI
Alcohol and drugs*	1937	36.9	0.62	35.6–38.1
Alcohol only	1832	34.9	0.62	33.7–36.1
Drugs	187	3.6	0.24	3.1–4.0
Drugs only	82	1.6	0.16	1.3–1.9
No AOD	3212	61.6	0.63	60.3–62.8

*Consists of women taking either just alcohol or alcohol and drugs.

**Table 3:** Alcohol, Smoking, and Substance Involvement Screening Test (ASSIST) scores for the sub-sample (*n* = 684).

	%	SE	95% CI
Lifetime use			
Tobacco	39.2	1.93	35.4–43.0
Alcohol	59.0	2.10	54.9–63.1
Cannabis	12.7	1.30	10.2–15.3
Methamphetamine	6.9	0.91	5.1–8.7
Frequency of use in past 3 months			
Tobacco			
Never used	61.1	1.93	57.3–64.9
Not in the past 3 months	10.5	1.35	7.9–13.2
Occasionally	6.7	1.09	4.6–8.9
Weekly/daily	21.6	1.69	18.3–24.9
Tobacco risk^†^			
Low risk	70.1	1.80	66.5–73.7
Medium risk	27.7	1.80	24.1–31.2
High risk	2.3	0.60	1.1–3.5
Alcohol			
Never used	41.3	2.10	37.1–45.4
Not in the past 3 months	21.9	1.75	18.5–25.4
Occasionally	26.9	1.97	23.1–30.8
Weekly/daily	9.9	1.35	7.2–12.5
Alcohol risk^††^			
Low risk	74.9	1.90	71.2–78.7
Medium risk	21.9	1.80	18.2–25.5
High risk	3.2	0.80	1.6–4.8
Cannabis			
Never used	87.3	1.31	84.7–89.8
Not in the past 3 months	9.9	1.25	7.4–12.3
Occasionally	1.7	0.39	0.9–2.4
Weekly/daily	1.2	0.30	0.6–1.8
Cannabis risk^†††^			
Low risk	96.4	0.60	95.3–97.5
Medium risk	3.2	0.50	2.1–4.2
High risk	0.4	0.20	0.0–0.9
Methamphetamine			
Never used	93.1	0.91	91.3–94.9
Not in the past 3 months	4.5	0.85	2.8–6.1
Occasionally	1.1	0.22	0.7–1.6
Weekly/daily	1.3	0.34	0.6–2.0
Methamphetamine risk^†††^			
Low risk	97.2	0.40	96.4–98.1
Medium risk	2.1	0.40	1.3–2.8
High risk	0.7	0.20	0.3–1.1
Any drug use (occasionally or weekly/daily)	4.7	0.60	3.5–5.9

^†^Tobacco risk 0–3 = low risk; 4–26 = medium risk; 27+ = high risk; ^††^Alcohol risk 0–10 = low risk; 11–26 = medium risk; 27+ = high risk; ^†††^Drug risk 0–3 = low risk; 4–26 = medium risk; 27+ = high risk.

**Table 4:** Alcohol, Smoking, and Substance Involvement Screening Test (ASSIST): substance involvement scores.

	Min.	Max.	Mean	SE	Median
Tobacco score	2	34	15.8	0.57	16.2
Alcohol score	2	39	12.4	0.56	10.8
Cannabis score	2	36	9.9	1.25	5.5
Methamphetamine score	2	36	12.8	1.58	8.9
Total illicit substance score	2	36	12.2	1.18	7.7

**Table 5:** Prevalence of alcohol and other drug use among the sub-sample (*n* = 684): Urinalysis.

	Response*	%	SE	95% CI
Lost samples		12.2	1.3	9.6–14.7
				
Positive for drugs	Yes**	18.9	1.6	15.8–22.1
	No	68.9	1.9	65.3–72.6
				
Met standardized drug cut-off	Yes	8.8	1.1	6.7–10.9
	No	79.1	1.6	75.9–82.2
				
Positive for alcohol	Yes***	19.9	1.7	16.6–23.1
	No	67.6	1.9	64.0–71.2
				
Met standardized alcohol cut-off	Yes	19.6	1.7	16.3–22.8
	No	67.9	1.8	64.3–71.5
				
Positive for either drugs or alcohol	Yes	33.5	2.0	29.7–37.4
	No	54.3	2.0	50.4–58.2
				
Met standardized cut-off for drugs or alcohol	Yes	25.8	1.8	22.2–29.4
	No	62.0	1.9	58.2–65.9
Polydrug use****	No drugs	68.9	1.9	65.3–72.6
	1 drug	15.8	1.5	12.9–18.8
	>1 drug	3.1	0.6	1.9–4.3

*34 women have no urine data and 61 have no alcohol and methamphetamine data due to the specimens having been inadvertently destroyed; **70 of these women were not classified as positive according to standardized cut-off levels; ***2 of these women were not classified as positive according to standardized cut-off levels; ****refers to the use of more than one illicit drug and excludes alcohol.
